# Whole-Genome Profiling of Endophytic Strain B.L.Ns.14 from *Nigella sativa* Reveals Potential for Agricultural Bioenhancement

**DOI:** 10.3390/microorganisms12122604

**Published:** 2024-12-16

**Authors:** Dimitra Douka, Tasos-Nektarios Spantidos, Polina C. Tsalgatidou, Panagiotis Katinakis, Anastasia Venieraki

**Affiliations:** 1 Laboratory of General and Agricultural Microbiology, Department of Crop Science, Agricultural University of Athens, Iera Odos 75, 11855 Athens, Greece; demyduke@gmail.com (D.D.); tasos_spad@hotmail.com (T.-N.S.); katp@aua.gr (P.K.); 2 Department of Agriculture, University of the Peloponnese, 24150 Kalamata, Greece; polina.tsalgatidou@go.uop.gr; 3 Laboratory of Plant Pathology, Department of Crop Science, Agricultural University of Athens, Iera Odos 75, 11855 Athens, Greece

**Keywords:** endophytes, *Bacillus halotolerans*, biocontrol, secondary metabolites, bacterial genome mining, biosynthetic gene clusters, medicinal plant endophytes, PGPRs, plant growth promotion, saline conditions

## Abstract

Endophytic microbes in medicinal plants often possess beneficial traits for plant health. This study focuses on the bacterial endophyte strain B.L.Ns.14, isolated from *Nigella sativa* leaves, which demonstrated multiple plant growth-promoting properties. In vitro tests showed that B.L.Ns.14 supports plant growth, colonization, and tolerance to abiotic stress. The strain also exhibited antifungal activity against phytopathogens such as *Rhizoctonia solani*, *Colletotrichum acutatum*, *Verticillium dahliae*, and *Fusarium oxysporum* f. sp. *radicis-lycopersici*. Whole-genome analysis, supported by ANI and dDDH values, identified B.L.Ns.14 as *Bacillus halotolerans*. Genome mining revealed 128 active carbohydrate enzymes (Cazymes) related to endophytism and biocontrol functions, along with genes involved in phosphate solubilization, siderophore and IAA production, biofilm formation, and motility. Furthermore, genes for osmolyte metabolism, Na+/H+ antiporters, and stress response proteins were also identified. The genome harbors 12 secondary metabolite biosynthetic gene clusters, including those for surfactin, plipastatin mojavensin, rhizocticin A, and bacilysin, known for their antagonistic effects against fungi. Additionally, B.L.Ns.14 promoted *Arabidopsis thaliana* growth under both normal and saline conditions, and enhanced *Solanum lycopersicum* growth via seed biopriming and root irrigation. These findings suggest that *Bacillus halotolerans* B.L.Ns.14 holds potential as a biocontrol and plant productivity agent, warranting further field testing.

## 1. Introduction

The safety of plant yield and the strengthening defense systems against biotic and abiotic stresses, in an ecological manner, is one of the greatest challenges in agriculture. Worldwide, biotic stress has been estimated to reduce the annual production of about 30% of crops, while 50% of arable land is expected to be affected by abiotic factors such as drought and salinity by 2050 [1,2]. Various approaches, such as the selection of tolerant varieties, molecular breeding, and genetic engineering, are proposed to improve the resistance of varieties to various stresses. However, most of these techniques are expensive, time-consuming, and unpopular in some places. Therefore, in order to improve plant health and productivity, it is imperative to develop simpler, more economical and environmentally friendly methods [2,3].

Plants are largely considered as meta-organisms where their growth, nutritional status, and ecological fitness are a result of the interactions between the plant and its microbiome (an assembly of microorganisms that live together in the inner/outer plant tissues and rhizosphere), influenced by environmental conditions [1,4]. Plant endobiome (endpophytic microbes) develop symbiotic relationships with the host and serve essential ecological functions. Endophytes inhabit a plant’s internal tissues (i.e., the endosphere) which provide a shelter to thrive from drastically variable external conditions [5,6,7]. In turn, endophytes promote the growth of plants via their ability to make essential nutrients bioavailable and the synthesis of phytohormones [8,9,10,11]. They also protect plants from pathogenic microorganisms through the occupation of the same niche within plants, the secretion of antimicrobial secondary metabolites, and the stimulation of host immunity [12,13,14]. Moreover, endophytes contribute to alleviation of plants in abiotic stress conditions through the induction of organic osmolyte production (such as trehalose, proline, glycine, and betaine), the enhancing of antioxidant defense system to scavenge reactive oxygen species (ROS), the mediation of hormonal profiles, and the maintaining of ionic homeostasis by controlling ion accumulation [15,16,17]. 

Research focused on the isolation and selection of promising endophytic species, such as bacterial endophytes, with beneficial characteristics in order to be exploited for the development of bioagent–inoculants [6,8]. Among the endophytic species, Bacillus bacteria are ubiquitous and emerged for their bioprospects as biostimulant and biocontrol agent candidates [18,19]. To date, numerous studies have shown that members of this genus promote plant growth and induce resistance/tolerance throughout the host plants to a wide range of biotic and abiotic stresses (drought, extreme temperatures, salinity, etc.), thereby leading to increased crop productivity. Their action includes the successful colonization ability, the synthesis of broad-spectrum bioactive compounds with antibiotic activity, siderophores, enzymes, phytohormones, and the regulation of their levels in plants, improving nutrient uptake and inducing plant systemic resistance [19,20,21,22,23]. Furthermore, *Bacillus* spp. exert great survival ability under harsh environmental conditions due to the formation of long-lived stress-tolerant endospores. Lastly, plant-associated Bacillus generally are recognized as safe (GRAS) for human health and the environment, thereby these characteristics make them an attractive option for the development of biological products to protect plants and enhance their productivity [18,20,21,22,23].

The relationship between hostplants and endophytes can be regarded as flexible and dynamically influenced by both biotic and abiotic factors, but also by host plant species and their characteristics, which can shape the community structure, composition, diversity and functions of endophytic species that can colonize a host plant throughout its life cycle [6,24,25,26,27]. According to literature reports, medicinal plants are considered as a rich harbor of diverse microbial endophytes endowed with beneficial properties [28,29]. *Nigella sativa*, commonly known as black seed, is an annual herbaceous flowering plant originating in the Mediterranean region. Black seed is used for therapeutic purposes due to its antimicrobial, antioxidant, and anti-inflammatory properties [30,31,32].

The current study aimed (a) to isolate endophytic bacteria from the medicinal plant *N. sativa* and determine the most promising antagonistic strain against several fungal pathogens, (b) to evaluate the selected strain in vitro for plant growth-promoting properties and to investigate its ability to alleviate abiotic stresses, (c) to examine its potential beneficial effect on *Solanum lycopersicum*, as well as on the model plant *Arabidopsis thaliana* in vitro under both saline and non-saline conditions, and (d) to perform whole-genome sequencing and genome mining analysis concerning the presence of genes and biosynthetic gene clusters involved in colonization, biological control, plant growth promotion, and abiotic stress tolerance.

## 2. Materials and Methods

### 2.1. Plant Sample and Bacterial Endophyte Isolation

The asymptomatic plant *N. sativa* was collected from an experimental field of the Agricultural University of Athens, Greece during June of 2016. The plant tissues (leaves, roots, flowers, and barks) were excised and subjected to surface sterilization according to Kusari et al. (2012) [33]. Excised tissues were washed in 70% ethanol for 1 min and in 5% (*v*/*v*) aqueous washing solution (commercial bleach and 0.1% Tween-20) (Merck KGaA, Darmstadt, Germany) for 3 min. The tissues were also immersed in 70% ethanol for 30 s and finally were rinsed 3 times in ddH_2_O. Aliquots of the last step of the protocol were plated in nutrient agar (NA, Condalab, Madrid, Spain) and incubated at 30 °C for 10 days. These plates served as control for the successful disinfection, regarding the presence or absence of microorganism growth colonies. The tissues were pulped by a mortar and pestle and plated on nutrient agar plates (NA, Condalab, Spain) amended with Cycloheximide (100 μg/mL) (Merck KGaA, Darmstadt, Germany) and incubated at 30 °C for 3 days. Intact surface sterilized tissues were also incubated by using a second control. Colonies were selected and purified in nutrient agar after 3 days of total incubation period. For each Petri dish evaluated, the colonies were selected according to their time of growth and morphology (color, size, and shape). Strains were kept as solid cultures on NA at 4 °C and then they were cryopreserved at −80 °C as a 40% glycerol stock for long-term storage.

### 2.2. Antifungal Activity

All the bacterial isolates were tested against four phytopathogenic fungi *Rhizoctonia solani*, *Fusarium oxysporum* f.sp. *radicis-lycopersici*, *Colletotrichum acutatum*, and *Verticillium dahliae* using dual culture assay in vitro on a NA medium (Condalab, Spain) in order to determine the most antifungal-effective strains. At a distance of 3 cm from the edge of the plate was placed a mycelial plug (size 6 mm) from a 10-day fungal culture grown on NA medium (Condalab, Spain). At the opposite diametrical point and at a distance of 3 cm from the edge of NA plates, an aliquot of 5 μL bacterial inoculums from an overnight Nutrient Broth (NB) culture (NB, Condalab, Spain) was inoculated. As control, we used fungal cultures on NA medium without bacterial treatment. All the treatments and the controls were incubated at 25 °C for 15 days. The experiment was performed 3 times. The antagonistic activity of bacterial treatment was evaluated by the measuring of the mycelial radius of each phytopathogenic fungus and calculating the bacterial inhibition index using the formula Ι% = (ρ1 − ρ2)/ρ1 × 100 [34].

### 2.3. Plant Growth-Promoting Traits

All the isolated bacterial endophytes were tested for siderophore production by CAS agar assay [35]. An aliquot of 10 μL of an NB overnight culture was spotted on the center of the plate and incubated at 30 °C for 3 days. Positive results were confirmed by the formation of an orange-yellow halo around the colony. The examination of precipitated phosphate solubilization was carried out by inoculating 10 μL of overnight bacterial culture on the Pikovskaya medium phosphate [36] and incubated at 30 °C for 7 days. The formation of a clear halo around the colony after incubation at 30 °C for 7 days showed the potential bacterial ability of phosphate solubilization. Protease production was tested by spotting 10 μL of an overnight bacterial culture on the plate on a CYEA medium (0.5% casein, 0.25% yeast extract, 0.1% glucose, and 1.5% agar) (Merck KGaA, Darmstadt, Germany) supplemented with skim milk powder (7%) and incubation [37]. The appearance of a clear halo around the colony after incubation at 30 °C for 3 days showed the positive result. Cellulase production was assayed by inoculating 10 μL of an overnight bacterial culture on CYEA medium (0.5% casein, 0.25% yeast extract, 0.1% glucose, and 1.5% agar) amended with 1% CMC (Carboxylmethyl cellulose, Alfa Aesar GmbH & Co KG, Ward Hill, MA, USA) [38]. The plate was flooded with Congo Red solution (1 mg/mL) (Merck KGaA, Darmstadt, Germany) and remained for 15 min. Then, the plate was flooded again with 1M NaCl solution to remove unbound Congo Red solution. A positive result was indicated by the formation of a clear halo around the colony after incubation at 30 °C for 3 days. Urease production was tested by spotting 10 μL of overnight bacterial on a Urea Base Christensen ISO 6579, ISO 19,250 (Condalab, Madrid, Spain) medium [39] and incubated at 30 °C for 2 days. The appearance of a pink halo around the colony showed ureolytic activity. Acetoin production was screened by the Voges–Proskauer test [40], where the positive result is linked with the appearance of a pink-red color in the liquid medium.

### 2.4. Motile Ability and Biofilm Formation

The ability to perform swarming and swimming motility was screened by inoculation of 5 μL and 3 μL, respectively, from a bacterial overnight NB culture on a NA medium with either 0.5% or 0.3% agar. The plates were incubated at 30 °C for 1 day, where the bacterial spread on the surface semi-solid medium indicated the motile ability. Biofilm formation was determined using the crystal violet staining assay in 96-well PVC plates [41]. An aliquot of 100 μL from a diluted bacterial overnight culture (1:100 dilution) was inoculated in wells (5 replicate wells). The 96-well plates with the treatments were incubated at 30 °C for 1 day, without agitation. Then, the plates were thoroughly washed 3 times with dH_2_O and 200 μL of 0.1% (*w*/*v*) crystal violet were added in the wells. The plates were incubated at room temperature for 30 min. The stain was discarded by washing and 200 μL of a solution (20% (*v*/*v*) ethanol and 80% (*v*/*v*) acetone) was added. The plates were incubated again at room temperature for 30 min to remove the biofilm formed by the stained cells from the walls of the wells. The presence of the purple-blue color is linked to the ability of biofilm formation. All the aforementioned assays were carried out three times in three independent experiments.

### 2.5. Abiotic Stress Tolerance Assay

The examination of survival ability under abiotic stress was carried out by inoculation-spot on NA medium plates amended with either 2.5% or 5% NaCl for salt stress conditions, as well as NA medium plates with either 2.5% or 5% polyethylene glycol (PEG3000) for drought stress conditions. For the inoculation, an aliquot 5 μL of 10^−1^ to 10^−5^ dilutions from a bacterial overnight NB culture was inoculated on the plates and incubated at 30 °C for 24 h. Similarly, the survival ability under different temperatures was determined by spotting of 5 μL from 10^−1^ to 10^−5^ dilutions on NA medium plates and incubating at 20 °C, 30 °C, and 40 °C for 24 h. 

### 2.6. Antibiotic Susceptibility Trait

The bacterial antibiotic susceptibility was evaluated by Kirby–Bauer antibiotic testing [42]. An aliquot 100 µL of overnight bacterial culture was plated on NA plates and left to dry in a sterilized laminar flow cabinet. Then, paper disks (diameter 6 mm) containing 20 μL of three tested concentrations (10, 30, and 50 μg/mL) from 6 commercial antibiotics, kanamycin, rifampicin, ampicillin, streptomycin chloramphenicol, and tetracycline, were placed on the surface of the medium. The plates were incubated at 30 °C for 48 h. The formation of a clear halo around paper disk indicated the susceptibility to specific antibiotic and concentration.

### 2.7. Plant Growth-Promoting Activity on A. thaliana Col-0 Seedlings In Vitro Under Saline and Non-Saline Conditions

For this experiment, the modified protocol of Palacio-Rodríguez et al. (2017) [43] was followed. The seeds of *A. thaliana* Col-0 were washed for 0.5 min in 70% ethanol and immersed for 1.5 min in washing solution (5% (*v*/*v*) aqueous solution of commercial bleach and 0.1% Tween20) (Merck KGaA, Darmstadt, Germany). The seeds were washed again for 30 s in 70% ethanol and finally rinsed in sterile dH_2_O. Sterilized seeds were placed on ½ Murashige and Skoog supplemented with vitamins (½ MS) (MS0222, Duchefa Biochemie, Haarlem, The Netherlands), and amended with 1.5% sucrose and 0.6% agar. Then, all plates were maintained at 4 °C for 2 days and were placed at an angle of 70° or horizontally (I-plate) in a growth chamber (22–25 °C, 16 h light: 8 h dark photoperiod) for three days. To evaluate the bacterial effect on plant growth under normal conditions, six emerged seedlings were transplanted on growth medium (½ MS with vitamins, 1% sucrose, and 0.8% agar). For saline conditions, the seedlings were applied on the MS agar plates where 100 mM NaCl were added. The inoculation spot was performed by 10 μL of bacterial inoculant (10^8^ CFU/mL) 3 cm under the root tips. As control, we used seedlings without bacterial treatment. All the plates were kept in the growth chamber at a vertical position. After nine days after sowing (DAS) the plantlets were evaluated for their fresh weight, the length of the primary root, and the number of the lateral roots.

The protocol which was used to examine the potential beneficial effect of the producing bacterial volatiles on plant growth was based on Asari et al., 2016 modified protocol [44]. For normal conditions, six growing seedlings per Petri dish were used and placed on one half of an I-plate with growth medium (½ MS with vitamins, 1.5% sucrose, and 0.8% agar). For saline conditions was used the same growth medium with 100 mM NaCl, respectively. Then, on the other half of the I-plates, bacterial inoculation spots were performed (3 spots of 20 μL of bacterial suspension 10^8^ CFU/mL). The plates were double-sealed with parafilm and placed in a horizontal position in the growth chamber. As control, we used seedlings without bacterial treatment. After 14 DAS, the plantlets were evaluated for their shoot fresh weight and the rosette diameter.

### 2.8. Plant Growth-Promoting Activity on Tomato Seeds and Plants 

The surface sterilization protocol of tomato seeds of var. Chondrocatsari was performed according to the following steps: sowing in 70% ethanol for 0.5 min, immersing in the washing solution (commercial bleach with 0.1% Tween 20) for 3 min, and thoroughly rinsing with ddH_2_O. The sterilized seeds were left to dry in a laminar flow cabinet and then they were incubated for one hour in a bacterial inoculum with two concentrations (10^6^ and 10^8^ CFU/mL) suspended with 1% CMC. As controls, tomato seeds without bacterial treatment, but only with 1% CMC solution, were used. All the seeds left aseptically dried again and were transferred to plates containing filter paper soaked in ddH_2_O. The plates were kept at 25 °C for 5 days in the dark. For the experiment, 3 biological replicates were performed with 15 seeds per replicate. The potential biopriming effect was studied by measuring the length of the primary roots that emerged from the germinated seeds, as well as by calculating the percentage of germination % according to the type GP% = (g/t) × 100, where g represents the number of seeds that germinated, and t represents the total number of seeds.

In addition, 2-day germinated seeds were obtained by seed-coating with 10^8^ CFU/mL and 1% CMC were sown in plastic plant pots (8 × 8 × 8 cm) containing a mixture of peat/perlite (5:1 ratio). After 15 days, bacterial inoculation was performed by applying an aliquot of 10 mL of bacterial suspension 10^8^ CFU/mL per pot. The plants were grown at 25 ± 5 °C in a photoperiod (14 h light/10 h dark) and watered 3 times a week with a specific volume of water. After 28 DAS, the potential plant growth effect was evaluated after measuring the fresh weight, the height, and the dry weight of the tomato shoots. For the experiment, 30 plants were used for each treatment and 3 biological replicates were performed. Plants without bacterial treatment were served as controls.

### 2.9. Phylogenetic Recognition Based on 16S rRNA Sequence 

The kit of Nucleospin^®^ Microbial DNA (Macherey-Nagel GmbH & Co. KG, Düren, Germany) was used to isolate bacterial DNA from an overnight bacterial culture. For amplification of 16S rRNA gene fragment, the following set of primers was used: F: 5′-AGAGTTTGATCCTGGCTCAG-3′ and R: 5′-ACGGCTACCTTGTTACGACTT-3′ [45]. BLASTn search for obtaining the 16S rRNA sequence was performed for genus-level recognition according to the phylogenetically closest strains deposited in GenBank.

### 2.10. Whole-Genome Sequencing

Genomic DNA was extracted from an overnight liquid culture of *Bacillus* strain B.L.Ns.14 using the PureLink^®^ Genomic DNA Mini Kit (Thermo Fisher Scientific, Carlsbad, CA, USA). The obtaining DNA was sequenced by SNPsaurus (Eugene, OR, USA) using an Illumina HiSeq 2000 platform. A library was generated using a Nextera XT DNA Library Prep Kit (Illumina, Inc., San Diego, CA, USA) and the sequencing was trimmed with BBDuk and then assembled with SPAdes-3.12.0 using default parameters [46]. The final de novo genome of strain B.L.Ns.14 (NCBI accession: JAEACL000000000) was assembled de novo in 10 scaffolds with a genome total size of 4,054,777 bp. Phylogenomic analysis of the strain was established by Type (strain) Genome Server (https://tygs.dsmz.de) accessed on 15 April 2024, and the tree was generated with FastME from Genome BLAST Distance Phylogeny (GBDP) distances [47]. The accurate phylogenetic taxonomy of the B.L.Ns.14 was carried out by calculating the orthologous average nucleotide identity (OrthoANI) and the digital DNA:DNA hybridization (dDDH) using the genome-to-genome distance calculator website service (GGDC 3.1) under the recommended formula. For ANI and dDDH, obtaining values are in accordance with species delineation threshold values that were suggested by default analysis (95–96% and 70%, respectively) [48,49].

The secondary metabolites were analyzed by antibiotics and a secondary metabolites analysis shell (antiSMASH) using the web server https://antismash.secondarymetabolites.org/. The adenylation and condensation structures of non-ribosomal polypeptide synthetases (NRPSs) and their amino acid sequence were identified using antiSMASH and the PKS/NRPS Analysis Web-tool (https://nrps.igs.umaryland.edu/).

The carbohydrate-active enzymes (CAZymes) were detected using the CAZy open database https://www.cazy.org/ and the ANnotation dbCAN3 web server (https://bcb.unl.edu/dbCAN2/). 

### 2.11. Statistical Analysis

Statistical analyses and plots were carried out using the GraphPad Prism program (Prism 10) (GraphPad Software, San Diego, CA, USA). The comparison of the bacterial treatment to the control was performed by two-tailed independent samples Student’s *t*-test (*p* < 0.05). Plots represent average values, error bars indicate the standard deviation, and asterisks the statistical differences.

## 3. Results

### 3.1. Isolation of Endophytic Bacteria from Nigella sativa

A total of 94 single bacterial colonies were isolated from flowers, leaves, barks, and roots of the medicinal plant *N. sativa*. These colonies were tested macroscopically and stereoscopically for their features. All bacterial isolates were massively screened for their antagonistic activity against the phytopathogenic fungus *R. solani* in a multiple-culture system in vitro. B.L.Ns.14 was the most promising strain, regarding the size of the inhibition zone, compared to the other endophytic isolates, so it was selected for further characterization.

### 3.2. Biocontrol Ability of Bacterial Endophyte B.L.Ns.14

**Figure 1:**
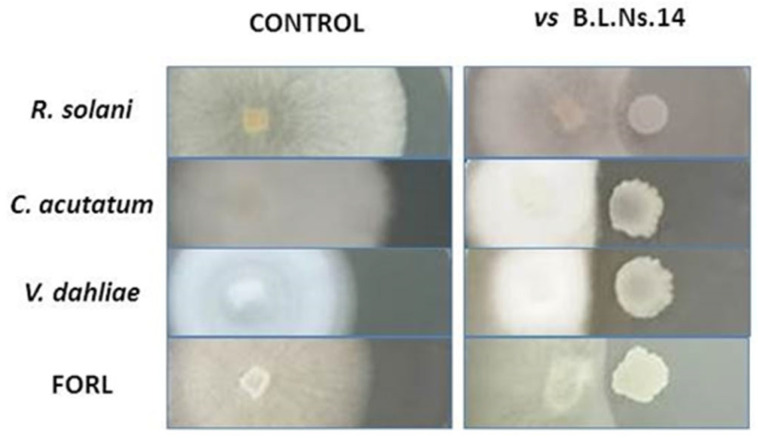
Illustration of antifungal activity of B.L.Ns.14 in vitro by using dual culture assay.

The strain B.L.Ns.14 was tested for its antagonistic activity through dual culture assay in vitro against a broad range of fungal plant pathogens, including *R. solani*, *C. acutatum*, *V. dahliae,* and *F. oxysporum* f.sp. *radicis-lycopersici* (FORL). The results reveal that the strain constrains the radial growth of all fungal plant pathogens (Figure 1, Table 1).

### 3.3. Plant Growth Promotion and Colonization Ability of Bacterial Endophyte B.L.Ns.14

**Figure 2:**
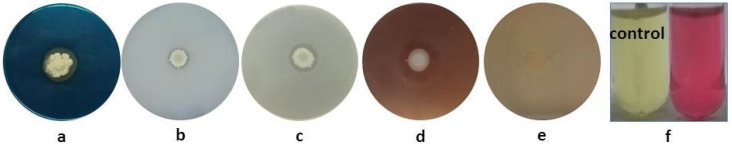
Plant growth-promoting traits of B.L.Ns.14; (**a**) siderophore production, (**b**) phosphate solubilization, (**c**) protease secretion, (**d**) cellulase secretion, (**e**) urease production, and (**f**) acetoin production.

**Figure 3:**
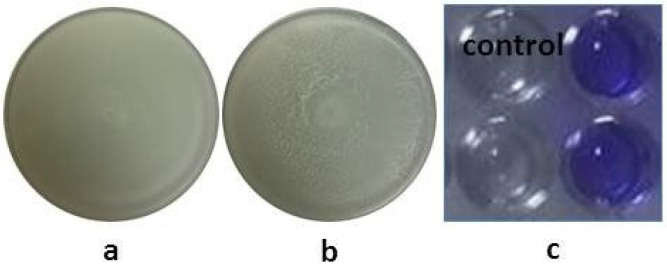
Colonization-related traits of B.L.Ns.14; (**a**) swarming motility, (**b**) swimming motility, and (**c**) biofilm formation ability.

In an in vitro screening for plant growth-promoting traits, B.L.Ns.14 showed positive results in acetoin production, chelation iron mobilization, and phosphate solubilization (Figure 2). Furthermore, it exhibited lytic enzymes production such as protease and cellulase, but not urease (Figure 2). Lastly, the excellent ability of the strain for swarming and swimming was observed, as well as biofilm formation in polystyrene wells (Figure 3).

### 3.4. Tolerance in Abiotic Stresses

**Figure 4:**
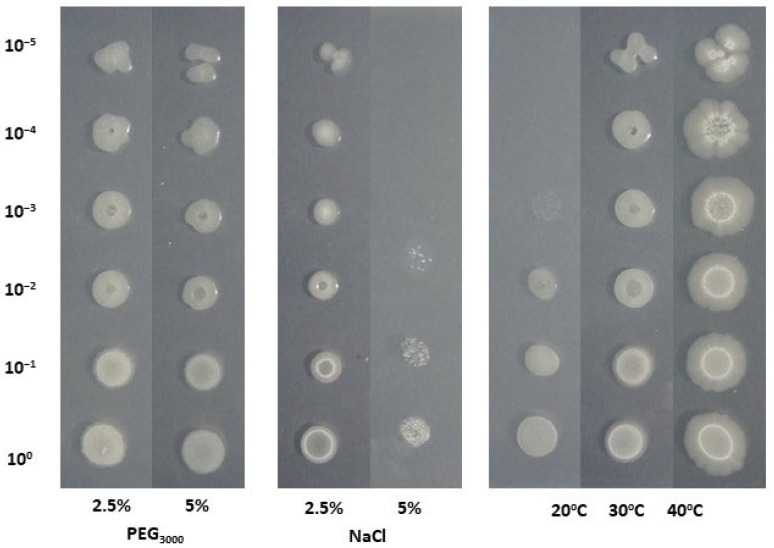
Survival ability of B.L.Ns.14 under drought and salt stress conditions, as well as under different temperatures.

Strain B.L.Ns.14 was examined for its tolerance under drought and salinity conditions in vitro. Regarding the drought stress assay, it was found that the strain showed an equally high survival capacity, since growth was observed at up to 10^−5^ dilution at both 2.5% and 5% PEG_3000_ concentrations. For the survival assay under salinity conditions, it was found that the strain B.L.Ns.14, showed an increased ability to survive in a concentration of 2.5% NaCl, where bacterial growth was detected up to 10^−5^ dilution, compared to the 5% NaCl concentration where it was grown up to 10^−2^ dilution. Lastly, bacterial growth was detected at up to 10^−5^ dilution at both 30 °C and 40 °C temperatures, but the strain was grown up to 10^−3^ dilution at 20 °C (Figure 4).

### 3.5. Antibiotic Susceptibility 

**Figure 5:**
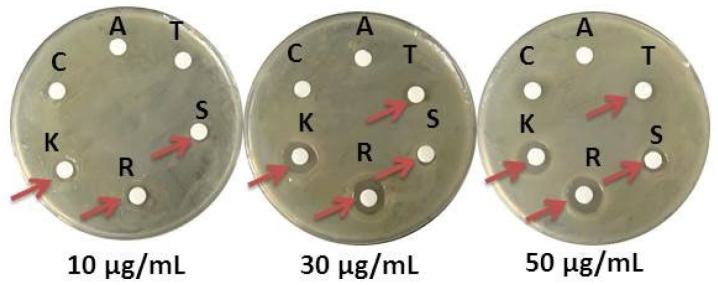
Antibiotic susceptibility test of B.L.Ns.14 by using six commercial antibiotics of three fixed concentrations (μg/mL); (A) ampicillin, (T) tetracycline, (S) streptomycin, (R) rifampicin, (K) kanamycin, and (C) chloramphenicol. The absence or presence of the clear zone around the soaked paper disks shows resistance or susceptibility of the strain. Red arrows indicate the susceptibility of the strain defined by the halo formed around the paper disks.

The antibiotic susceptibility of B.L.Ns.14 was examined by KB assay by using six commercial antibiotics (i.e., ampicillin, tetracycline, streptomycin, rifampicin, kanamycin, and chloramphenicol) of three fixed concentrations (10, 30, and 50 μg/mL). The appearance of a clear halo around the filter paper indicates the sensitivity of the strain to the respective antibiotic and concentration. The B.L.Ns.14 showed complete resistance to ampicillin and chloramphenicol, while sensitivity was observed to tetracycline (30 and 50 μg/mL), as well as kanamycin, rifampicin, and streptomycin (10, 30, and 50 μg/mL) (Figure 5).

### 3.6. Phylogenetic Recognition of the Bacterial Endophyte Strain B.L.Ns.14

**Figure 6:**
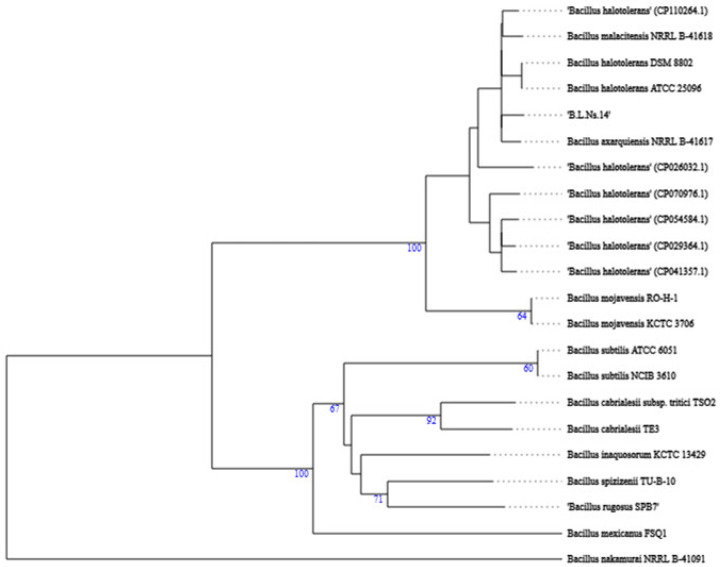
Sequence-based phylogenomic tree constructed on TYGS (https://tygs.dsmz.de/) depicting the position of bacterial strain B.L.Ns.14 relative to other phylogenetically close species. The tree was generated with FastME from Genome BLAST Distance Phylogeny (GBDP) distances [49]. The numbers above the branches are GBDP pseudo-bootstrap support values >60% from 100 replications. The branch lengths are scaled in terms of GBDP distance formula d5 and the tree was rooted at the midpoint. The accession numbers of genome sequences are listed in parentheses.

Based on the in vitro screenings, strain B.L.Ns.14 gathers enough biocontrol and plant growth-promoting features, thus further characterization was needed. BLASTn analysis of the 16S rRNA sequence and comparison to type strains of the GenBank database of NCBI revealed that this strain is more closely related to the Bacillus species, with a 98.29% identity to the *Bacillus rugosus* strain sz30 (NCBI accession; OR826039.1). To achieve verified classification, the whole-genome sequence of strain B.L.Ns.14 was compared with those of other Bacillus strains using the Type (strain) Genome Server and revealed the close affiliation to the *B. halotolerans* species (Figure 6). In addition, the OrthoANI and dDDH values between B.L.Ns.14 and nine *B. halotolerans* strains ranged between 97.83–99.18% and 80.8–94.1%, respectively, and were higher than the published species thresholds (95–96% for OrthoANI, and 70% for dDDH) [48,49] (Table 2). Therefore, the results of the analysis from the ANI and dDDH methods are in agreement and consistent with those from the genome-based phylogenetic analysis, so strain B.L.Ns.14 was classified as *B. halotolerans*.

### 3.7. Investigation of Secondary Metabolites Gene Clusters in the B.L.Ns.14 Genome

The strain B.L.Ns.14 (*B. halotolerans*) was studied to find biosynthetic gene clusters of secondary metabolites with antibiotic and/or biocontrol activity. After AntiSMASH analysis, it was found that in this strain there were 12 individual BGCs in 11 biosynthetic regions, with region 4.3 comprising 2 classes of BGCs (Table 3). All the above BGCs are involved in the production of known compounds as well as unknown or others not listed in the MIBIG database.

**Figure 7:**
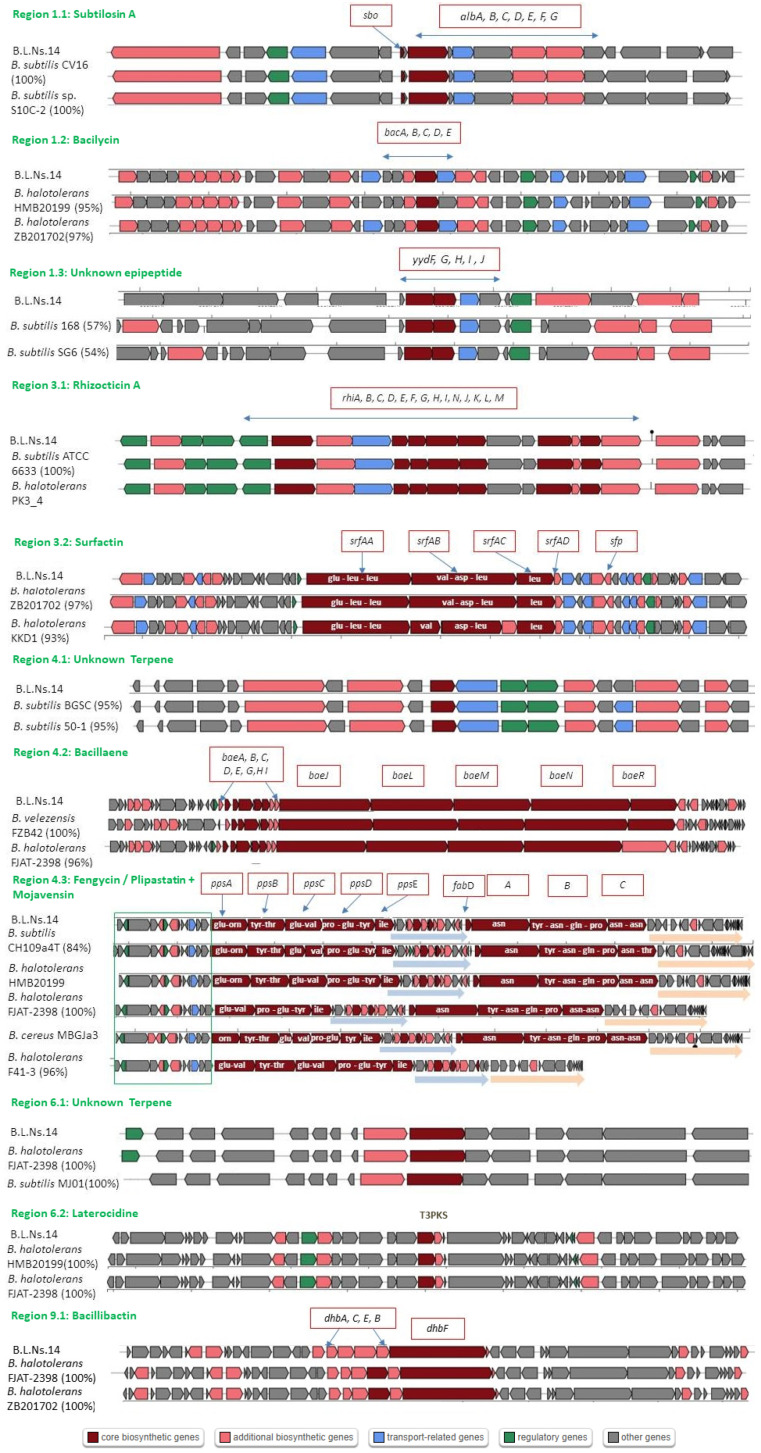
Results of antiSMASH analysis of the *B. halotolerans* B.L.Ns.14 genome. Illustration of detecting genomic regions where biosynthetic gene clusters of secondary metabolites are located. The closest core biosynthetic gene clusters of known and unknown BGCs according to the MIBiG database are depicted along with some of the best hits in ClusterBlast. Core biosynthetic genes of the cyclic lipopeptides and the aminoacid sequences are reported, as well as the gene similarity percentage given by antiSMASH.

Out of 12 BGCs, only 3 (i.e., 2 terpene BGCs, and 1 epipeptide BGC) were found to be incompatible with other known compounds in the databases despite being common to other *B. halotolerans* strains showing high gene similarity. Specifically, after ClusterBlust analysis, the terpene BGC of regions 4.1 showed 95% homology with other strains of *Bacillus subtilis* (strains 50-1, BGSC etc.), and the terpene BGC of region 6.1 showed 100% homology with *B. halotolerans* FJAT-2398 and *B. subtilis* MJ01. Additionally, in region 1.3, a epipeptide BGC was found which was not associated with any known compound, but ClusterBlust analysis showed 57% homology with *B. subtilis* ydFGHIJ gene cluster encoding the post-ribosomal synthesized peptide YydF, predicted to be the epipeptide precursor (Figure 7).

Regarding the known compounds as shown in Figure 7, the biosynthetic clusters showed a high homology of 86–100% with the deposited BGCs in the MIBIG database and shared identical gene organization with homologous BGCs found in several *B. halotolerans*, *Bacillus velezensis*, and *B. subtilis* strains and related to subtilosin A, bacilycin, bacillaene, bacillibactin, and surfactin with the reference strains (Table 3). Particularly, the bacillibactin BGC which was found in the B.L.Ns.14 genome (region 9.1) showed 100% homology with *B. halotolerans* (strains ZB201702, FJAT-2398 etc.), and the bacillaene BGC of region 4.2 showed 100% homology with *B. velezensis* FZB42.

Additionally, in region 4.3 where 2 biosynthetic gene clusters were found, one related to BGC fengycin (100% homology), while the other appeared as an iturin complex showing 100% homology with mycosubtilin, 60% with bacillomycin D, and 44% with iturin A. However, the predicted amino acid sequence showed that the specific biosynthetic gene cluster encodes mojavensin synthases, while BGC fengycin is related to the plipastatin compound. BlastN searches using mojavensin BGC and fengycin/plipastatin BGC as a probe revealed that not all *B. halotolerans* strains contained both 2 BGCs as in the case of B.L.Ns.14. Specifically, strain F41-3 genome harbor only the biosynthetic cluster of fengycin/plipastatin, in contrast, HMB20199 genome harbor both 2 BGCs in their genome (Figure 7).

Another interesting observation was the presence of Rhizocticin A BGC in the B.L.Ns.14 genome, located in the 3.1 region. The core biosynthetic genes of Rhizocticin A in the genome of strain B.L.Ns.14 also shared an extensive sequence identity in *Bacillus subtilis* ATCC6633 (homology 90.86%) and *B. halotolerans* PK3_4 (98.92%) as well (Figure 7).

Lastly, in region 6.2 was detected a PKS type III BGC showing 5% similarity with laterocidine BGC. According to ClusterBlust analysis, the laterocidine gene cluster of strain B.L.Ns.14. showed high gene similarity (100%) to a PKS gene cluster found in several *B. halotolerans* strains (strain FJAT-2398, HMB20199 etc.) (Figure 7).

### 3.8. The Bacterial Endophyte Β.L.Ns.14 Strain Possesses Genes Involved in Biological Control, Plant Growth Promotion, Colonization, and Abiotic Stress Tolerance

Genome mining analysis of Β.L.Ns.14 also revealed the existence of genes related to biological control, plant growth promoting, and colonization properties. Particularly, genes encoding 128 active carbohydrate enzymes (Cazymes) were identified in the genome of B.L.Ns.14 through the open database cazY and server dbCAN3 () (https://bcb.unl.edu/dbCAN2/, accessed on 15 April 2024). Among the 128 Cazymes, 50 belonged to families of glycoside hydrolases (GHs), 37 to glycosyltransferases (GTs), 7 to lyases (PLs), 15 to polysaccharide esterases (CEs), 3 to growth factors (AAs), and 16 in families of carbohydrate-binding proteins (CBMs). Of the above Cazymes, 36 (28.125%) possess an amino-terminal end of the signal peptide that mediates the export of the protein through the cytoplasmic membrane (Supplementary Table S1).

Genes involved in phosphorus solubilization (phoA, phoB, phoD, and phy), indoleacetic acid biosynthesis (trpA, trpΒ, trpC, trpD, and trpE), and the production of acetoin and 2, 3 butanediol (bdhA, budA, alsS, ilvN, ilvB, AcuA, AcuB, and AcuC) were also found. Moreover, genes related to flagellum synthesis and function (flgB, flgC, fliE, fliG, flgG, fliL, fliM, flip, fliQ, flhA, flhB, flit, fliS, fligK, and flgL), biofilm formation (csrA, wecB, remA, YlbF bslA, bslB, tasA, and rpoN), exopolysaccharides production (epsG), as well as genes related to swarming motility (SwrA, SwrB) and chemotaxis (CheA, CheC, CheD, CheV, CheW, and CheY) were detected. Lastly, genes detected for abiotic stress tolerance were found, including genes related to ion homeostasis (NhaC) and biosynthesis of osmolytes such as proline (proA, proB, and proC), glycine/betaine (OpuAC, OPuD), glutamate (gltB, gltD), glutamine (glnA), as well asgenes encoding stress response proteins (DnaK, GroES, GroEL, DnaK, and dnaJ) and spermidine (speA, speB, and speE).

### 3.9. The Β.L.Ns.14 Strain Was Able to Boost the Growth of the Model Plant A. thaliana Under Normal and Saline Conditions

**Figure 8:**
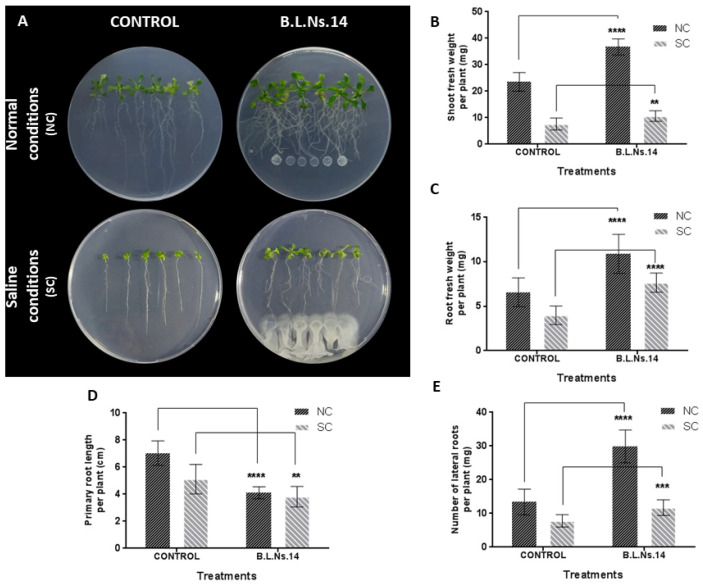
The beneficial activity of Β.L.Ns.14 on *A. thaliana* Col-0 seedlings in vitro, under normal and saline conditions (100 mM NaCl). (**A**) Representative images of the seedlings for the two treatments under normal (NC) and saline (SC) conditions; (**B**) shoot fresh weight of the seedlings (mg) (*n* = 12); (**C**) root fresh weight of the seedlings (mg) (n = 12); (**D**) primary root length (cm) (n = 12); and (**E**) total lateral root number (n = 12). Data represent the mean (SD) of seedlings from one representative experiment. Asterisks indicate statistically significant differences after *t*-test analysis (**, *p* < 0.01, ***, *p* < 0.001, ****, and *p* < 0.0001).

As can be seen, Β.L.Ns.14 possesses potential plant growth promoting and abiotic stress tolerance traits (properties based on genomic analysis and in vitro experiments). Thus, in the next step we investigated the plant growth-promoting effect on *A. thaliana* seedlings growing on MS agar medium under normal and saline conditions. In order to determine whether the effect is a result of strain’s production of diffusible and or volatile compounds, Petri dishes with transplanting seedlings were used in I-plates and mono plates, as well. It was revealed that bacterial inoculant applied below the root tip proved effective to elevate the growth parameters tested. Particularly, after 9 days of co culture under normal conditions, plant biomass and root surface area increased. In addition, morphological alteration of root system architecture was detected, which encompasses the shortening of the primary root length and the triggering of lateral root formation, resulting in clumped root phenotype. Similar observations were made under saline conditions where Β.L.Ns.14 was capable to alleviate the salt stress and enhance the plant biomass (Figure 8).

**Figure 9:**
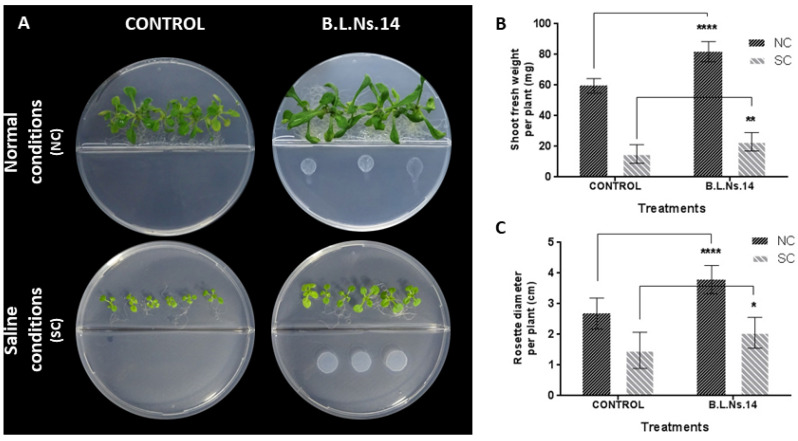
The beneficial activity of Β.L.Ns.14 on *A. thaliana* Col-0 seedlings in vitro via volatile emission, under normal and saline conditions (100 mM NaCl). (**A**) Representative images of the seedlings for the two treatments under normal (NC) and saline (SC) conditions; (**B**) shoot fresh weight of the seedlings (mg) (n = 12); and (**C**) rosette diameter (cm) (n = 12). Data represent the mean (SD) of seedlings from one representative experiment. Asterisks indicate statistically significant differences after *t*-test analysis (*, *p* < 0.1, **, *p* < 0.01, and ****, *p* < 0.0001).

Lastly, shoot fresh weight and leaf area of the seedlings were also increased through volatile compounds after 14 days of co-culture in I-plates under normal and saline conditions, respectively (Figure 9).

### 3.10. The Β.L.Ns.14 Strain Beneficially Affected the Growth of Solanum lycopersicum var. Chondrokatsari Messinias

**Figure 10:**
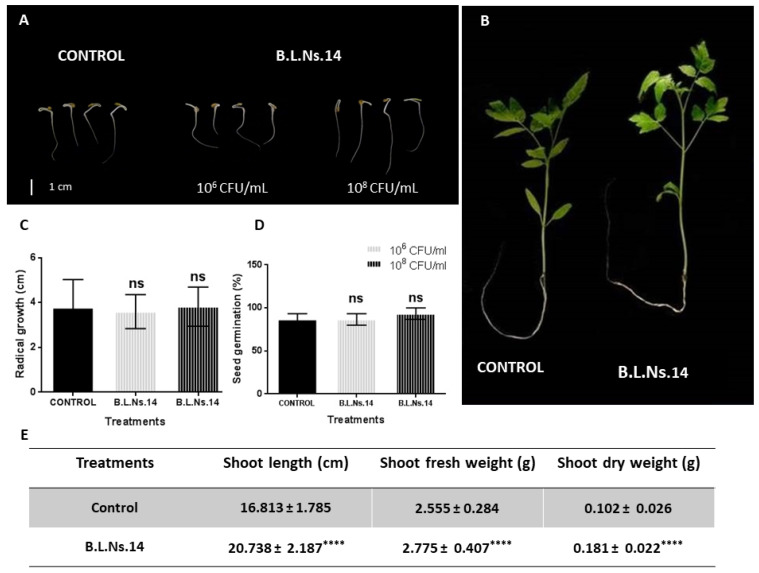
Plant growth-promoting effect of Β.L.Ns.14 on *S. lycopersicum* var. Chondrokatsari Messinias; depiction of tomato seedlings (**A**) treated by seed biopriming method (scale bar = 1 cm) after 5 days; (**B**) treated by seed biopriming and root irrigation after 4 weeks; (**C**) radical growth (cm) emerged from bioprimed seeds from 3 replicates after using inoculants with bacterial suspensions (10^6^ CFU/mL and 10^8^ CFU/mL), each containing 15 seeds (n = 3); (**D**) germination (%) of bioprimed seeds with bacterial suspensions (10^6^ CFU/mL and 10^8^ CFU/mL), each containing 15 seeds (n = 3); and (**E**) data of shoot length (cm), shoot fresh weight (gr), and shoot dry weight (gr) of tomato seedlings (n = 30) after 4 weeks emerged from bioprimed seeds and root irrigation with bacterial suspension (10^8^ CFU/mL) in pots. Data represent the mean (SD) of seedlings and asterisks indicate statistically significant differences after *t*-test analysis (ns, non-significant; ****, *p* < 0.0001).

The plant growth-promoting effect of the strain was evaluated by the seed bioprimingon tomato seeds. Bacterial inoculant was performed by using CMC (1%) and two testing cell suspensions (10^6^ and 10^8^ CFU/mL). Seeds of native Greek variety *Solanum lycopersicum* var. Chondrokatsari Messinias were used for the bacterial treatment and incubated for 5 days. The determination of the bacterial effect was made by measuring the length of the primary root and seed germination percentage. The results reveal that the strain applied to the seeds was capable to little increase the growth parameters, but the differences compared to the control were not significant (Figure 10A,C,D). 

Following the seed biopriming assay, we wished to examine the potential plant growth-promoting effect of the strain on the tomato plants growing in pots by using a combination of the seed coating and root irrigation techniques. For the experiment, bacterial inoculant with 10^8^ CFU/mL cell suspension was performed. After recording shoot length, shoot fresh weight, and dry weight of treated plants for a 4-week growth period, we observed that the Β.L.Ns.14 strain proved effective to enhance all growth characteristics tested compared to the control plants (Figure 10B,E).

## 4. Discussion

Endophytic bacteria colonize the interior tissues of plants without causing any significant damage but offer various beneficial effects to their hosts. The genotype, morphology, life history, and health status of plants, as well as the external conditions can influence the composition and distribution of their endophytic bacterial community [6,25]. Medicinal plants are considered as a rich harbor of promising endophytic bacteria which have the functions of promoting the growth of host plants, protecting from various pathogens, and enhancing the stress resistance of the host plants, while they have the potential to regulate the synthesis of various bioactive metabolites in plants and to metabolize medicinal compounds [6,29,51]. To the best of our knowledge, this is the first study where endophytic bacteria isolated from *N. sativa* were characterized for biocontrol, stress resistance, and plant growth-promoting features, where the B.L.Ns.14 strain gathered the most promising beneficial functions.

In this study, culturable endophytic bacteria were isolated from the flowers, leaves, bark, and root of the medicinal plant *N. sativa*. Among the isolates, B.L.Ns.14 displayed strong antagonistic activity against fungal pathogens in vitro. According to the biochemical traits tested, the isolate showed positive results in essential characteristics for plant development, tolerance to abiotic stress, and biocontrol activity. Particularly, the strain was capable to produce acetoin, mobilize chelate iron, solubilize precipitated phosphorus, and secrete lytic enzymes (cellulase and protease). Increased survival ability under harsh conditions in vitro such as high temperature, salinity, and drought was also noticed. Moreover, the strain proved effective in colonization-related traits such as swarming and swimming motility, as well as biofilm formation ability, contributing to the successful bacterial approach and adherence on the plant tissues [52,53].

Many studies have reported the isolation of endophytic bacteria from various medicinal plants, which have been extensively studied for their beneficial properties, with Bacillus emerging as one of the most commonly detected genera [29,54,55,56,57]. The rapid development of gene sequencing technology and bioinformatic means brought progressing steps to the field of microbiology research in general and specifically the endophytes’ examination, allowing the in-depth analyses of genetic material regarding the accurate taxonomy [58,59] and the possessing of biosynthetic potential [59,60,61,62,63,64].

Thus, bacterial isolate was subjected to a whole genome sequence for elucidating the underlying genomic information related to biological control and plant growth-promoting functions, but also to achieve accurate taxonomic recognition. The genome-wide phylogenomic tree (which was constructed) showed that the strain was closely phylogenetically affiliated (located in the same branch) with other *B. halotolerans* strains. This finding was also confirmed by dDDH and ANI values, suggesting that B.L.Ns.14 is classified as *B. halotolerans*.

Furthermore, antiSMASH analysis of the B.L.Ns.14 genome revealed the existence of putative secondary metabolites’ BGCs related to the production of antimicrobial compounds with broad-spectrum activity against plant pathogens. Twelve BGCs across 11 genomic regions were identified, covering a considerable part (13.61%) of the strain’s genome. Among these gene clusters, three BGCs were also detected that were unknown according to the MiBIG database, indicating that the strain has the genomic capacity to synthesize new antimicrobial substances that need further study. Indeed, further analysis of a BGC found in region 1.3 (locus 442,018–463,719 nt) revealed that the core genes organization of this biosynthetic gene cluster is identical to BGC, which encodes a well characterized post-translationally modified peptide (RiPP) biosynthesis. Thus, strain B.L.Ns.14 might have the potential to synthesize the epipeptide YydF, a very affective bacteriocin against Gram-positive bacteria [65,66,67]. 

In addition, a putative PKS gene cluster which was located in region 6.2 was observed and showed 5% similarity to laterocidine, a macrocyclic lipopeptide which selectively combats Gram-negative bacteria and is produced by strains of B. laterosporus [68]. 

The remaining eight BGCs were detected with high homology and related to known antimicrobial compound synthesis of non-ribosomal peptides, polyketides, and RiPPs. Subtilosin A is an antimicrobial peptide belonging to sactipeptides, a subcategory of RiPPs, produced by the soil bacterium *Bacillus subtilis*, and displays bactericidal activity against a diverse range of bacteria, including *Listeria monocytogenes* [69,70]. It is encoded by the operon composed of the genes sboA and albABCDEFG, as they were identified in B.L.Ns.14. The sboA gene is responsible for encoding the 43-amino-acid precursor of subtilosin A and the downstream albABCDEFG genes related to the processing and modification of the precursor peptide, the formation and export of the mature peptide, as well as the immunity to subtilosin A [71,72,73]. Bacillaene is a linear polyketide/nonribosomal peptide produced by Bacillus strains, known for its antibacterial activity against both Gram-positive and Gram-negative bacteria by inhibiting prokaryotic protein synthesis. It is synthesized by the trans-acyltransferase polyketide synthetase and is encoded by bae operon [74,75]. Bacillibactin is a microbial catecholate siderophore, which is a non-ribosomal peptide that is synthesized. It is encoded by dhb operon consisted of dhbACEBF genes and plays an important role in the biocontrol activity of *Bacillus* spp. against plant pathogens, both through antibiosis iron and scavenging [76,77]. Bacilysin is an antimicrobial dipeptide acting against phytopathogenic bacteria and fungi. It is synthesized by NRPs and is encoded by bac operon. It contains an L-Ala residue at the N-terminus and an L-anticapsin at the C-terminus, which interferes with glucosamine synthetase disrupting peptidoglycan synthesis in bacterial pathogens or mannoprotein biosynthesis in fungal pathogens [69,78].

Cyclic lipopeptides (CLPs) are considered the spearhead of the antibiotic arsenal of *Bacillus* spp. due to the wide range of bioactivities they display, including biosurfactant, antimicrobial, antiviral, anticancer, and immunosuppressive activity, as well as they are involved in root colonization and in systemic stimulation of host plant immune system [79,80]. Bacillus’ CLPs are synthesized by NRPSs and include three main families (surfactins, fengycins, and iturins). Their chemical structure varies by CLP family, but generally includes a peptide backbone consisting of seven or ten amino acids and a β-hydroxy or β-amino fatty acid with alkyl chains to form a macrocyclic [80]. Surfactin is encoded by the srfAA-srfAD gene cluster and exhibits antibacterial and antiviral activities, as well its role is critical for biofilm formation, swarming motility, and root colonization [79,81]. Plipastatin belongs to the fengycin family and is encoded by the ppsABCDE gene cluster, as we observed in the B.L.Ns.14 genome [79]. Plipastatin, known for its potent antifungal activity against a variety of filamentous fungi, is commonly associated with strains of both *B. subtilis* and *B. halotolerans* [82,83]. The plipastatin BGC of the B.L.Ns.14 genome was adjacent to an iturinic-type BGC. Based on the predicted amino acid sequence of the final product, this BGCs is proven to encode for the synthetases of mojavensin. Mojavensin belongs to the iturin family and shows antifungal activity [84,85]. The presence of fengycin/plipastatin and mojavensin BGCs were also reported by Thomloudi et al. (2021) and Tsalgatidou et al. (2022) [86,87] in *B. halotolerans* strains, where it was observed that mojavensin BGC is not present in all strains of this species, but is gained or lost by horizontal transfer. For instance, the recently published genomes of *B. halotolerans* F41-3 [82] and KKD1 strains [83], as well as the PK3_4 strain contain only the plipastatin BGC but not the entire mojavensin BGC, unlike with other *Bacillus* spp. strains phylogenetically more distant [86,87,88]. Our findings after comparing the genomic regions between B.L.Ns.14 and *Bacillus cereus* MBGJa3, which both contain mojavensin BGC, confirm the aforementioned observation. Possible prospects correlated with discovered metabolites are the confirmation of the production and functionality of the predicted metabolites using analytical techniques such as LC-MS to isolate and identify the compounds synthesized in vitro and in planta and to clarify the exact role and effectiveness of each metabolite in controlling specific pathogens or modulating plant responses, and additionally, to utilize the metabolites for developing efficient biological control strategies against plant pathogens by introducing and testing B.L.Ns.14 in planta and exploring formulations combining these metabolites to optimize biocontrol efficacy, and furthermore, to understand how the metabolites suppress pathogens and promote plant health by investigating mechanisms of action and exploring synergistic effects among the metabolites, and of course, to analyze ISR pathways triggered by metabolites such as bacilysin and surfactin.

Notably, according to our results, the mojavensin BGC was not the only one that might be gained or lost through horizontal gene transfer, as it was absent from the genome of most of the *B. halotolerans* strains (e.g., *B. halotolerans* F41-3). A BGC with 100% homology to Rhizocticin A was detected in the B.L.Ns.14 genome, according to the MIBiG database. Rhizocticin A is a phosphonate oligopeptide antibiotic with antagonistic activity against fungi and nematodes, as well. It is consisted by an arginine linked with the fungitoxic L-2-amino-5-phosphono-3-cis-pentanoic acid (L-APPA) which interferes with threonine metabolism in fungal cells and inhibits cell growth [69,89,90]. It is synthesized by NRPSs through a non-thiotemplate mechanism and is encoded by the rhi gene cluster, first reported in *B. subtilis* ATCC6633 [91].

Apart from the unique antibiotic repertoire detected in B.L.Ns.14 genomic comparisons with other *B. halotolerans* strains reported, the biocontrol capacity of the strain is also enhanced by the presence of lytic enzymes. CAZyme analysis showed the existence of 128 putative gene-encoding enzymes, some of which participate in biocontrol activity. For example, chitinase (GH18) and chitosanase (GH46) potentially combat phytopathogenic fungi directly or indirectly by eliciting plant defense reactions after chitinolytic activity and chitosan fragments are released [92,93]. Moreover, α-amylase (GH13), β-xylosidase (GH43), β-glucosidase (GH1, GH3), endoglucanase (GH51), and pectate lyases that were detected might be involved in the endophytic colonization ability of the strain [94].

Genome mining results provide insights into PGP properties and colonization ability of the strain, many of which were already confirmed by in vitro evaluation. The B.L.Ns.14 genome possesses genes that participated in phosphorus solubilization and in the production of siderophore bacillibactin, facilitating the uptake of phosphorus and iron by plants. Genes involved in the production of volatile plant growth regulators and plant defense elicitors acetoin and 2,3 butanediol are contained, as well as genes responsible for IAA.

It is known that most promising bioagents cannot propagate under stressful conditions, so the ability to withstand harsh environments is critical to microbial survival and, hence, their functionality [83]. In the current study, genomic analysis enlightened genetic features about abiotic stress tolerance of the strain. Particularly, we detected genes DegS and DegU linked with signal transduction, suggesting that our strain is able to sense different abiotic stresses and respond at an early stage [95]. Moreover, genes encoded sodium; proton antiporters were present and contributed to maintaining cellular homeostasis under salt conditions by exchanging protons for sodium ions [83]. Genes involved in the synthesis of important osmoprotectants were discovered, such as glycine/betaine, proline, glutamate, and glutamine, aiding to osmotic stress regulation [95,96,97,98,99]. Lastly, the B.L.Ns.14 genome contains genes encoding stress response proteins that enable the strain to tolerate heat and cold shock stress [100].

Environmental stressors, such as salinity, are a major constraint affecting crop growth and productivity worldwide. The detrimental effects of high salt concentrations on plants are well-documented, leading to decreased water uptake, impaired nutrient acquisition, as well as osmotic and oxidative stress [101,102,103]. PGP bacteria emerged as promising candidates for promoting plant growth and inducing stress tolerance [104,105,106]. In accordance to literature, Bacillus species confer salt stress tolerance in plants through various mechanisms; by producing EPS, activating the plant’s anti-oxidation system, increasing total soluble sugar content, decreasing Na+ accumulation, and modulating hormone signaling pathways [95,107,108,109,110,111,112].

In our study, in silico and in vitro evaluation showed that strain B.L.Ns.14 gathers several PGP characteristics, making it a promising microbial biostimulant. Subsequently, the application of the strain to the model plant *A. thaliana* under normal conditions, with two different inoculation techniques (at a distance and on I-plates) showed a significant increase in the growth characteristics tested and, therefore, expanded the knowledge of its PGP ability so far. Our results are in line with previous related studies by Thomloudi et al. (2021) [86] and Tsalgatidou et al. (2022; 2023) [87,88], where *B. halotolerans* strains beneficially affected plant growth of *A. thaliana* seedlings, possibly through diffusible and/or volatile compounds that may have altered root architecture, leading to an increase in total fresh weight and leaf area. 

Furthermore, recent studies also demonstrated that application of *B. halotolerans* encourages wheat growth under saline conditions [83]. Based on our results, inoculation of the strain below the root tip was able to colonize the roots and increase the growth parameters examined in *A. thaliana* seedlings compared to untreated seedlings, indicating the adaptation of the plant to salinity. In addition, changes in the root architecture of the seedlings were observed in this case as well. These changes include the increase in lateral root number and total root surface area, leading to better plant access to nutrients. The plant’s salt tolerance findings might be attributed to the combined effect of the strain’s exopolysaccharide production, volatile emission, and phytohormone regulation. Exopolysaccharides produced during biofilm formation and colonization surround roots, helping maintain ionic balance in the plant under salinity, and enhanced soil accumulation improves nutrient absorption and water retention, properties that lead to mitigation of salt stress in plants [113,114,115,116]. According to Liu et al., 2017, the EPS from Bacillus FZB42 alleviate salt-stress by binding and inhibiting Na+ uptake in Arabidopsis, and eventually reduce Na+ concentration in the plant [117]. Moreover, the regulation of the levels of specific phytohormones is an important factor in plant tolerance to salt stress. In the study of Zhang et al., 2022, it was found that the up-regulation of genes encoding IAA, as well as the concentration of IAA in seedling roots caused by endophytic B. cereus, led to the enhancement of *A. thaliana* seedlings’ response to salt stress [118]. Therefore, one possible explanation for the seedling’s salt tolerance provoked by the inoculation of the strain B.L.Ns.14 is probably related to the auxin contents induction in *A. thaliana* tissues, since the root phenotype alteration which was observed is considered as an “auxin-dependent phenomenon” [119,120,121].

Last but not least, the role of volatile emissions from B.L.Ns.14 on the induction of salt tolerance could not be ignored. Especially, when the strain inoculated in the one compartment, it provoked significant increase in the shoot fresh weight and leaf area of the seedlings placed on the other side of I-plates. The beneficial effect of VOCs from *Bacillus* spp. on plant growth under salt stress may be attributed to the activation of ion transporters (e.g., tissue-specific regulation of the sodium transporter HKT1 and NHX1 antiporter), resulting in the decrease in Na+ accumulation throughout the whole plant, as well as to the modulation of hormonal pathways, the activation of the anti-oxidation system, and the increase in total soluble sugar [108,122]. In addition, the profile analysis of the emitted VOC blend from the known beneficial strain *B. subtilis* GB03, grown under salt conditions, showed the appearance of several compounds. Among these, acetoin emerged as the major one, showcasing its critical role in the salt stress adaptation of Mentha piperita plants [123].

Considering the plant growth effect of the strain B.L.Ns.14 on the model plant *A. thaliana*, we also suggested the evaluation of the strain’s potential on other plants (e.g., tomato plants). The application of PGP bacteria can be conducted through various techniques, including seed biopriming, foliar, root, and soil application [124,125,126,127], and even in a combination of these [124]. Therefore, seeds of *S. lycopersicum* var. Chondrokatsari Messinias were treated by B.L.Ns.14 through biopriming and in a combination of biopriming and root irrigation of growing plantlets in pots. The beneficial results of seed biopriming are widely known, conferring multiple advantages in plant health and development including the improvement of agronomical characteristics (such as seed germination and seedling vigor), as well as helping the plants to control abiotic and biotic stresses [128,129]. 

The individual seed-coating application of the B.L.Ns.14 strain caused an increase in seed germination and radical growth after 5 days of observation, but in a non-significant way. In a similar study by Thomloudi et al. 2021 [86], *B. halotolerans* Hil4 achieved earlier germination instead of the overall germination by significantly increasing the germination percentage of the tomato seeds at 3 days, but not at 8 days of incubation, boosting also other morphological characteristics. Nevertheless, it should be noted that the ability of great seed germination induction is a result of certain strains’ ability [130,131] or bacterial activity emerged under stress conditions such as drought [132] and saline conditions [130]. On the other hand, the combining application of the B.L.Ns.14 strain by biopriming and root irrigation was revealed as more effective, since it significantly enhanced the shoot length, as well as the fresh and the dry shoot weight (approximately up to 1.77-fold), compared to the untreated tomato plants. In agreement with our findings, Cabra Cendales et al., 2017 [133] reported that the combined application of the strain Bacillus pumilus GIBI 206, directly in the seeds and by peat impregnation, boosted the length and the fresh weight of stem and root in 37-day-old tomato seedlings. 

The positive effects of Bacillus strains’ application on crop plants have been evaluated by several researchers, who reported their great contribution to plant yield improvement [134,135], as well as to the management of severe diseases from plant pathogens [136,137,138,139,140] and the harmful impact of abiotic stresses [95,141,142,143,144,145]. As can be seen, the application of the *B. halotolerans* B.L.Ns.14 strain in field experiments and the thorough analysis of the multiple PGP modes of action that are activated, contributing to integrated plant protection from diverse factors, could be the subject of future studies. 

## 5. Conclusions

In the current study, we endophytically isolated the bacterial strain B.L.Ns.14 from the leaves of *N. sativa*, which was classified as *B. halotolerans* based on phylogenomic analyses. The results of whole genome mining and in vitro evaluation contributed to gaining comprehensive background information on the strain’s potential properties associated to plant growth promotion, biological control activity, and abiotic stress tolerance. In planta experiments revealed the strain’s efficacy in boosting the growth of unstressed *A. thaliana*, as well as in ameliorating the harmful effect of the salt-stressed *A. thaliana* seedlings. In addition, the combining bacterial application on *S. lycopersicum* also demonstrated positive results in the agronomical traits tested. Finally, the findings of this study can be helpful for the development of a novel biological agent that could be used as biopesticide and/or biostimulant, even under harsh external conditions. However, more research is needed to explore the functional validation of genes related the aforementioned bioprospects and the conduction of future field experiments, as well.

## Figures and Tables

**Table 1:** Antifungal effect ofB.L.Ns.14 against *R. solani*, *C. acutatum*, *V. dahliae,* and *F. oxysporum* f. sp. *radicis-lycopersici* (FORL).

	Mycelial Radius (cm) ^1^		Index Inhibition (%) ^1^
Pathogens	Control	Dual Culture	
*R. solani*	3.39 ± 0.76	3.09 ± 0.49 ****	64.95 ± 5.58
*C. acutatum*	3.55 ± 0.44	1.68 ± 0.19 ****	52.82 ± 5.49
*V. dahliae*	2.74 ± 0.19	1.33 ± 0.15 ****	51.58 ± 5.57
*FORL*	3.09 ± 0.49	0.98 ± 0.17 ****	68.34 ± 5.60

^1^ Data represent the mean (SD) of mycelial radius and asterisks indicate statistically significant difference after *t*-test analysis (****, *p* < 0.0001).

**Table 2:** Phylogenetic recognition of endophytic bacterial strain B.L.Ns.14 based on closely related bacterial strains belonging to *Bacillus halotolerans* strains, after average nucleotide identity by orthology (OrthoANI) and digital DNA-DNA hybridization (dDDH) analysis. ANI values 95–96% and dDDH values 70% are considered threshold values for species separation. Type strains are indicted by the letter ^T^.

	orthoANI%	dDDH%
Β.L.Ns.14	100	100
*B. halotolerans* strain PK3_4 (CP026032.1)	97.83	80.8
*B. halotolerans* strain ZB201702 (CP029364.1)	97.93	81.8
*B. halotolerans* strain F41-3 (CP041357.1)	97.9	81.6
*B. halotolerans* strain KKD1 (CP054584.1)	97.88	81.7
*B. halotolerans* strain MBH1 (CP070976.1)	97.85	80.9
*B. halotolerans* strain HMB20199 (CP110264.1)	99.18	93
*Bacillus axarquiensis* strain NRRL B-41617 ^T^ (NZ_LPVD00000000.1) *	99.08	92.7
*Bacillus halotolerans* strain ATCC 25096 ^T^ (NZ_LPVF01000014.1)	99.1	94.1
*Bacillus malacitensis* strain NRRL B-41618 ^T^ (NZ_LPVE00000000.1) *	99.1	91.9

* The strains *B. axarquiensis* NRRL B-41617 and *B. malacitensis* NRRL B-41618 are classified as *B. halotolerans* [50].

**Table 3:** Genomic regions and detected secondary metabolites BGCs of the genome of strain *B. halotolerans* B.L.Ns.14 by using the antiSMASH server and MIBiG database; NRPS = non-ribosomal peptide synthetase and PKS = polyketide synthetase.

Region	MIBIG	Locus	Most Similar Known Cluster	Predicted Biosynthetic Gene Clusters	Metabolite
1.1	BGC0000602 (100%)	133,397–155,008	sbo-alb	Sactipeptide	Subtilosin A
1.2	BGC0000888 (100%)	166,972–208,390	bac	Other	Bacilycin
1.3	-	442,018–463,719	-	RiPP	Epipeptide
3.1	BGC0000926 (100%)	36,618–57,033	rhi	Phosphonate	Rhizocticin A
3.2	BGC0000433 (86%)	194.176–259,571	srf	NRPS	Surfactin
4.1	-	205,412–226,218	-	Terpene	-
4.2	BGC0001089 (100%)	825,111–940,119	bae	NRPS, transAT-PKS	Bacillaene
4.3	BGC0001103 (100%)	995,601–1,125,433	myc	NRPS, transAT-PKS	Mycosubtilin *
	BGC0001095 (100%)		fen	NRPS	Fengycin **
6.1	-	14,331–36,229	-	Terpene	-
6.2	BGC0000867 (5%)	91,630–132,727	-	T3PKS	Laterocidine
9.1	BGC0000309 (100%)	50,663–102,437	dhb	NRPS	Bacillibactin

* Refers to mojavensin due to the predicted amino acid sequence. ** Refers to plipastatin due to the D configuration of Tyr9.

## Data Availability

All 16S rRNA gene sequences’ accession numbers are available in the NCBI database. The *Bacillus halotolerans* strain B.L.Ns.14 whole genome projects is available in the NCBI database under the accession numbers JAEACL000000000 (GenBank), SAMN16949413 (BioSample) and PRJNA681329 (BioProject).

## Supplementary Materials

The following supporting information can be downloaded at: https://www.mdpi.com/article/10.3390/microorganisms12122604/s1, Table S1. Description of CAZymes gene families in the genome of *B. halotolerans* Β.L.Ns.14.
