# Supplementary material for: Whole-Genome Profiling of Endophytic Strain B.L.Ns.14 from Nigella sativa Reveals Potential for Agricultural Bioenhancement
Source: Microorganisms. 2024 Dec 16;12(12):2604. doi: 10.3390/microorganisms12122604 (PMC11678546; doi:10.3390/microorganisms12122604)
Supplement: Supplementary file 1 [file microorganisms-12-02604-s001.zip › microorganisms-3356634-supplementary.pdf]

Article

Whole-Genome Profiling of Endophytic Strain B.L.Ns.14 from *Nigella sativa* Reveals Potential for Agricultural Bioenhancement

Dimitra Douka [1], Tasos-Nektarios Spantidos [1], Polina C. Tsalgatidou [2], Panagiotis Katinakis [1] and Anastasia Venieraki [3,*]

[1] Laboratory of General and Agricultural Microbiology, Department of Crop Science, Agricultural University of Athens, Iera Odos 75, 11855 Athens, Greece; demyduke@gmail.com (D.D.); tasos_spad@hotmail.com (T.-N.S.); katp@aua.gr (P.K.)
[2] Department of Agriculture, University of the Peloponnese, 24150 Kalamata, Greece; polina.tsalgatidou@go.uop.gr
[3] Laboratory of Plant Pathology, Department of Crop Science, Agricultural University of Athens, Iera Odos 75, 11855 Athens, Greece
* Correspondence: venieraki@aua.gr

Supplementary Table S1. Description of CAZymes gene families in the genome of *B. halotolerans* B.L.Ns.14.

CAZyme Categories	Families	Activity	Gene copy numbers
**Glycoside hydrolases (GHs)**	GH1	β-glucosidase 4	**4**
	GH3	β-glucosidase 1	**1**
	GH4	maltose-6-phosphate glucosidase 4	**4**
	GH5	Cellulase	**1**
	GH11	endo-β-1,4-xylanase	**1**
	GH13	α-amylase	**6**
	GH16	Xyloglucan	**1**
	GH18	chitinase	**4**
	GH23	lysozyme type G	**4**
	GH26	β-mannanase	**2**
	GH30	endo-β-1,4-xylanase	**1**
	GH32	invertase	**2**
	GH42	β-galactosidase	**2**
	GH43	β-xylosidase	**4**
	GH46	Chitosanase	**2**
	GH51	Endoglucanase	**2**
	GH53	endo-β-1,4-galactanase	**1**
	GH65	α,α-trehalase	**1**
	GH68	Levansucrase	**1**
	GH73	lysozyme	**2**

	GH105	unsaturated rhamnogalacturonyl hydrolase	**2**
	GH109	α-N-acetylgalactosaminidase	**1**
	GH171	peptidoglycan β-N-acetylmuramidase	**1**
			**Total=50**
**Glycosyltransferases (GTs)**	GT1	UDP-glucuronosyltransferase	**3**
	GT2	cellulose synthase	**15**
	GT4	sucrose synthase	**6**
	GT5	glycogen glucosyltransferase	**1**
	GT8	α-1,3-galactosyltransferase	**1**
	GT26	UDP-ManNAcA	**1**
	GT28	1,2-diacylglycerol 3-β-galactosyltransferase	**3**
	GT35	glycogen	**1**
	GT51	murein polymerase	**3**
	GT83	undecaprenyl phosphate-α-L-Ara4N: 4-amino-4-deoxy-β-L-arabinosyltransferase	**2**
	GT151	murein polymerase	**1**
			**Total=37**
**Polysaccharide esterases (CEs)**	CE4	acetyl xylan esterase	**6**
	CE6	acetyl xylan esterase	**1**
	CE7	acetyl xylan esterase	**1**
	CE9	N-acetylglucosamine 6-phosphate deacetylase	**1**
	CE12	pectin acetylesterase	**3**
	CE14	N-acetyl-1-D-myo-inosityl-2-amino-2-deoxy-α-D-glucopyranoside deacetylase	**2**
	CE19	pectin methylesterase	**1**
			**Total=15**
**Pectate lyases (PLs)**	PL1	pectate lyase	**2**
	PL3	pectate lyase	**1**
	PL9	pectate lyase	**1**
	PL11	rhamnogalacturonan endolyase	**2**
	PL26	rhamnogalacturonan exolyase	**1**
			**Total=7**
**Growth factors (AAs)**	AA4	vanillyl-alcohol oxidase	**1**
	AA6	1,4-benzoquinone reductase	**1**
	AA10	lytic polysaccharide monooxygenases	**1**
			**Total=3**
**Carbohydrate-binding proteins (CBMs)**	CBM34	Binding proteins (GH18,	**1**
	CBM48		**1**
	CBM50		**11**

	CBM63	GH19, GH23, GH24, GH25 and GH73) in carbohydrate like cellulose and glucomannan (CBMs)	**1**
	CBM66		**1**
	CBM68		**1**
			**Total=16**
